# Obstacles to patient inclusion in CPR/DNAR decisions and challenging conversations: A qualitative study with internal medicine physicians in Southern Switzerland

**DOI:** 10.1371/journal.pone.0282270

**Published:** 2023-03-22

**Authors:** Michele Bedulli, Ilaria Falvo, Paolo Merlani, Samia Hurst, Marta Fadda

**Affiliations:** 1 Internal Medicine Service, Ospedale Regionale di Lugano, Ente Ospedaliero Cantonale, Lugano, Switzerland; 2 Institute of Public Health, Università della Svizzera italiana, Lugano, Switzerland; 3 Intesive Care Unit, Ospedale Regionale di Lugano, Ente Ospedaliero Cantonale, Lugano, Switzerland; 4 University Hospital Geneva, University of Geneva, Geneva, Switzerland; 5 Institute for Ethics, History and the Humanities, University of Geneva, Geneva, Switzerland; Medical University of Vienna, AUSTRIA

## Abstract

Despite cardiopulmonary resuscitation (CPR) and do-not-attempt-resuscitation (DNAR) decisions are increasingly considered an essential component of hospital practice and patient inclusion in these conversations an ethical imperative in most cases, there is evidence that such discussions between physicians and patients/surrogate decision-makers (the person or people providing direction in decision making if a person is unable to make decisions about personal health care, e.g., family members or friends) are often inadequate, excessively delayed, or absent. We conducted a study to qualitatively explore physician-reported CPR/DNAR decision-making approaches and CPR/DNAR conversations with patients hospitalized in the internal medicine wards of the four main hospitals in Ticino, Southern Switzerland. We conducted four focus groups with 19 resident and staff physicians employed in the internal medicine unit of the four public hospitals in Ticino. Questions aimed to elicit participants’ specific experiences in deciding on and discussing CPR/DNAR with patients and their families, the stakeholders (ideally) involved in the discussion, and their responsibilities. We found that participants experienced two main tensions. On the one side, CPR/DNAR decisions were dominated by the belief that patient involvement is often pointless, even though participants favored a shared decision-making approach. On the other, despite aiming at a non-manipulative conversation, participants were aware that most CPR/DNAR conversations are characterized by a nudging communicative approach where the physician gently pushes patients towards his/her recommendation. Participants identified structural cause to the previous two tensions that go beyond the patient-physician relationship. CPR/DNAR decisions are examples of best interests assessments at the end of life. Such assessments represent value judgments that cannot be validly ascertained without patient input. CPR/DNAR conversations should be regarded as complex interventions that need to be thoroughly and regularly taught, in a manner similar to technical interventions.

## Introduction

Do Not Resuscitate (DNR) or Do Not Attempt a (cardiopulmonary) Resuscitation (DNAR) is a medical order precluding cardiopulmonary resuscitation (CPR) in the event of cardiac and/or respiratory arrest. The use of DNAR orders has been increasing over the last decades and decisions regarding CPR and DNAR orders are increasingly considered an essential component of hospital practice [1]. Research shows that most patients would like to be consulted on these decisions [2, 3]. Furthermore, there is consensus that inclusion of patients in CPR/DNAR conversations can advance patient-centered care [4]. However, there is evidence that the necessary DNAR discussions between physicians and patients or surrogate decision-makers are often inadequate, excessively delayed, or absent [5]. This holds the risk of failing to adequately fulfill the two main intended purposes of DNAR orders, namely to act in accordance with patients’ (presumed) wishes and interests and prevent interventions that are considered “medically ineffective”, “medically inconsequential” or “medically harmful” with regards to the established goals of care [6].

Several reasons have been reported why physicians may avoid or be reluctant to hold CPR/DNAR conversations. Studies show that these conversations are extremely demanding for patients, surrogate decision-makers, and healthcare providers alike, and a potential source of conflict [5, 7]. Roadblocks include barriers related to patient and family factors (e.g., patients’ difficulty in accepting a poor prognosis or understanding treatment options, disagreement about goals of care, and lack of advance directives and/or decision-making capacity) [8], deficits in clinicians’ skills (e.g., feeling unprepared when conducting code status discussions due to lack of communication or negotiation skills) [9–11], clinicians’ beliefs (e.g., the belief that the patient is not in imminent danger of death or judgments regarding patients’ quality of life or prognosis) [12] and their anticipated emotions (e.g., feeling guilty for causing patient’s psychological distress or fear of litigation) [13]. Other obstacles include system/institutional limitations such as time constraints, lack of an appropriate opportunity to discuss end-of-life issues, and disagreement on who should be responsible for discussing DNAR issues [5, 14]. However, there is evidence that, even when DNAR/CPR discussions are initiated, physicians tend to conceal or manipulate certain information about the procedure and outcomes of CPR [15], resulting in patients having misconceptions regarding their likelihood of survival as well as the quality of the outcomes of CPR [16].

In Switzerland, the Swiss Academy of Medical Sciences (SAMS) published guidelines on CPR/DNAR decisions in 2021 [17]. According to these guidelines, CPR/DNAR decisions should be addressed carefully and at an early stage, i.e. before the acute situation arises that may lead to a cardiac arrest, and they should involve both the evaluation of the patient’s initial medical position and the determination of the patient’s wishes, goals, and expectations [17]. While patients should be actively involved in the decision-making process, the guidelines also recommend that whether the question of CPR is addressed more or less explicitly depends on the age, health status, and prognosis of the patient [17]. The public health crisis caused by COVID-19 reinforced physicians’ ethical obligations to protect the health of the public and to prudently steward limited resources. In April 2020, the American Medical Association (AMA) stated that it would be ethically justifiable for physicians to withhold CPR without seeking patients’ agreement during a public health crisis, as the goal is to maximize benefit (and minimize harm) for the greatest number of patients [18]. According to the AMA, the level of risk of exposure to health care personnel involved in the resuscitation and the risks that caregivers might in turn pose to others if infected are conditions that would make it ethically justifiable to place a DNAR order without patient involvement in the decision [18].

Pre-pandemic evidence from German-speaking Switzerland shows that more than 60% of patients did not recall having had a code status discussion during their hospital stay and more than 70% of physicians defined a DNAR status without prior discussion with the patient [19]. A survey of residents in charge of 206 patients including DNAR and CPR orders in the French-speaking region of Switzerland showed that participants cited the patients’ or their family’s resuscitation preferences as a major justification for placing a DNAR order in 18% of cases [20]. Participants justified patients’ inclusion in or exclusion from DNAR/CPR discussions using arguments based on respect for patient autonomy and decision-making capacity, as well as arguments based on their own clinical assessment of the patient [12]. Pre-pandemic evidence from the Italian-speaking region of Switzerland on physicians’ reasoning regarding CPR/DNAR decisions, and on the extent to which, why and how they include patients in these decisions, is currently missing. At a policy level, a decision support tool is available to physicians working in the four main hospitals in Ticino which clarifies the circumstances under which patients should be included in CPR/DNAR decisions (Ente Ospedaliero Cantonale’s internal document “D-SAN-004/B”). According to this tool, patient inclusion in the discussion is not essential if (1) the patient does not have functional limitations or major comorbidities, or if (2) the patient is approaching death. In the first case, the tool demands that a CPR order be given unless the patient explicitly refused cardiopulmonary resuscitation, while a DNAR code status should be included and implemented in the second case. Conversely, patient inclusion in the CPR/DNAR discussion is essential in all “intermediate situations”, e.g., with patients affected by comorbidities for whom death is not impending and for whom CPR attempts may bring some benefit.

In this latter setting, we conducted a study to qualitatively explore physician-reported CPR/DNAR decision-making approaches and CPR/DNAR conversations with patients hospitalized in the internal medicine wards of the four main hospitals in Ticino. In particular, the study has the objective of identifying possible tensions between the desired and actual decision-making approaches and conversations, accounting for the specificities of the current public health emergency caused by COVID-19. A tension can be defined as a situation that requires making a decision between two or more moral imperatives, neither of which is satisfactory or preferable, and where choosing one potentially results in transgressing the other(s) [21]. We decided to focus on Ticino and the hospital setting because evidence shows that advance directive completion in the Italian-speaking regions of Switzerland is at 17.9%, suggesting that the majority of CPR/DNAR decisions are addresses in the acute setting of the hospital [22].

## Methods

### Study design

We conducted a qualitative study using focus group interviews to explore resident and staff physicians’ decision-making approaches and practices of DNAR/CPR conversations with patients in the four main hospitals in Ticino, the Italian-speaking Canton of Switzerland. Focus groups are a qualitative method in which a trained moderator interviews a small group of research participants simultaneously. For this study, we decided to employ focus group interviews because this is a rapid and convenient method to collect data from several individuals concurrently (particularly in light of physicians’ demanding schedules), but also because this method capitalizes on study participants’ communication exchanges in order to generate richer data [23]. In a focus group, participants are encouraged to discuss a variety of topics and exchange opinions among each other. For this reason, focus groups are particularly useful to understand participants’ perspectives on the research topic, their knowledge and experiences, and can be used to explore not only what people think but also how and why they think that way [24]. While we initially opted for face-to-face focus group interviews to be held in person, several potential participants manifested difficulties in reaching the university facilities at the scheduled time. For this reason, a decision was made to conduct the focus group interviews online on Zoom (Zoom Video Communications, Inc., San Jose, CA, USA).

### Sampling and recruitment

To be included in the study, participants had to be employed as either resident or staff physician in the internal medicine department of one of the four main hospitals in Ticino (Bellinzona, Locarno, Lugano or Mendrisio). We included participants with at least six months of postgraduate clinical experience to increase the chances that participants had confronted with the issues of CPR/DNAR. We excluded medical students and physicians with less than six month clinical experience. Recruitment was conducted in two phases. In a first phase, the study was officially presented by MB at a colloquium attended by resident and staff physicians employed in the various departments of internal medicine in Ticino in May 2021. In a second phase, following the study presentation, MB sent an e-mail to potential participants (N = 135) inviting them to take part to the study. The e-mail included information on the study and an explanation of the voluntarily nature of participation. Importantly, participants were reassured that their identifying information would not be shared with anyone employed by the hospital, but only retained by the study team at the Università della Svizzera italiana (MF and IF). To take part to the study, participants had to fill out an online form on Qualtrics® (Qualtrics, Provo, UT, USA) which asked them their availability for the interview and a number of questions such as name/surname, gender, age, employment type (resident vs. staff), hospital of employment, medical specialization, years of clinical experience after obtaining the medical degree, any postgraduate training in clinical ethics, any experience in departments other than internal medicine, e-mail address and their availability. This information allowed us to allocate participants to four focus groups that were homogeneous in terms of employment type but heterogeneous in terms of age, gender, and hospital of employment.

### Data collection

We conducted four online focus groups on Zoom between the 12^th^ and 16^th^ of July, 2021. Three focus groups were held with resident physicians and one with staff physicians. Interviews lasted between 42 and 60 minutes. The focus groups were moderated by MF, a female postdoctoral researcher in biomedical ethics with extensive training in qualitative research methods, and co-moderated by IF, a female research assistant with a background in cognitive psychology and extensive training in qualitative data collection and analysis. The moderators introduced themselves at the beginning of each focus group. Neither the moderator nor the co-moderator had any working relationships with the study participants. This allowed to reduce any social desirability bias in participants’ reports.

During the interview, which was recorded following participants’ oral informed consent, we employed an interview grid developed *ad hoc* for the study purposes (S1 Table). Questions aimed to elicit participants’ broad definition of advance care planning, their specific experiences in making a decision on and discussing CPR/DNAR with patients and their families (including barriers, facilitators, ethical justifications for inclusion/exclusion of patients in/from the CPR/DNAR discussion, and perspectives on both current practices and desired ones), the stakeholders (ideally) involved in the discussion and their responsibilities. At the end of each focus group, participants were asked about the reasons for participating, their participation experience, and any additional comments. This debriefing phase served to identify and mitigate potential stressful effects following the focus group discussion as participants were asked to reflect on sensitive topics such as the end-of-life.

### Data analysis

The interviews were transcribed and anonymized by IF within two weeks after data collection. Shortly after each focus group, a debriefing between MF and IF occurred in which thematic insights were shared and noted in document words file placed before each transcript interview for following analysis. Subsequently, MB and MF performed a thematic analysis on the transcripts according to the analytical approach developed by Braun and Clarke [25]. After individually reading and coding the transcripts, MB and MF convened together and compared their codes to identify similarities and resolve discrepancies, develop broader thematic categories and subcategories, and highlight meaningful quotes. Finally, a report describing the study results was shared with the rest of the team for feedback. During both the data collection and the analysis phases, the team reasoned on their role in the interview encounter and the interpretation of the data, identified possible biases linked to professional or personal experiences with the end-of-life, and reflected on solutions to mitigate them. No transcripts were returned to participants for feedback. No software was used to manage the data. This manuscript adheres to the consolidated criteria for reporting qualitative research (COREQ) [26].

### Ethics approval and consent to participate

The study protocol was submitted to the Ethics Committee of the Canton of Ticino (ID Req-2021-00293) which established that the protocol did not require ethics approval, since the project does not fall under Article 3 of the Swiss Human Research Act. All participants provided oral informed consent to participate in this study.

## Results

### Characteristics of the sample

The final sample included 19 participants from the four main public hospitals in Ticino, of which 10 were women (see Table 1 for participants’ characteristics). Of the 153 potential participants invited in the study, 34 accessed the invitation survey and 30 provided their contact information. Response rate was RR = 12.4%. No reason was provided for non-participation. Participants’ mean age was M = 33.1 years (SD = 5.3). Mean age was M = 31.1 years (SD = 3.8) among residents and M = 39.2 years (SD = 4.6) among staff physicians. Among participants, 14 were resident physicians and 5 were staff physicians. Residents varied in the number of years of clinical experience, with most (n = 7) reporting 3–4 years of experience More than three quarters of the total sample (n = 15) did not have any postgraduate training in clinical ethics. The 4 participants reporting previous postgraduate training in clinical ethics included residents only.

**Table 1:** Participants’ characteristics (N = 19).

Characteristics	n (%)
Gender	
Female	10 (53%)
Male	9 (47%)
Age (years: mean ± SD)	
Residents	31.1 ± 3.8
Staff physicians	39.2 ± 4.6
All	33.3 ± 5.3
Role	
Resident	14 (74%)
Staff physician	5 (26%)
Years of clinical experience (residents only)	
1–2	3 (21%)
3–4	7 (50%)
5–6	4 (29%)
Post-graduate training in clinical ethics*	
No	15 (79%)
Yes (≥ 1)	4 (21%)

*No staff physician reported any post-graduate training in clinical ethics

### General description of the extracted themes

The analysis of the transcripts yielded three main themes. The first refers to the tension that participants reported between the ideal decision-making approach regarding CPR/DNAR and advance care planning, more generally, and the actual approach to patient involvement they experienced, which is described as being largely dominated by the belief that patient involvement is often pointless. Participants reported a number of justifications for this tension and proposed practical solutions to address it. The second theme refers to the tension between what participants regarded as ideal CPR/DNAR conversations with patients and how these actually unfold in their clinical experience; participants reported that they find themselves inevitably nudging patients rather than eliciting their preferences, an approach they justify through multiple explanations and for which they suggest a number of alternative practices. The third theme refers to the structural causes of this twofold tension: these include patient-provider-, policy-, culture-, and religion-related factors. Exemplary quotes from the focus group interviews can be found in Table 2.

**Table 2:** Exemplary quotes from the focus group interviews.

Domain	Theme	Sub-theme	Quote*	#
Decision-making				
	The ought			
		Anticipate	It is the watchword, it is what we have to do during the day. Anticipate, anticipate. (FG1)	1
			Discussing it now and not waiting until the last moment to do things like advance directives regarding the patient’s conception of life. These things should be done as early as possible, or at least introduced. (FG1)	2
			Always at the bases: anticipate everything. It may not be the solution to everything, but it is a good help. (FG1)	3
			Thinking in advance about extreme situations, and then saying yes or no to the type of treatment outcome we are willing to accept. (FG2)	4
			In the ideal world, no one should get to the age of ninety without knowing what kind of death one would want. (FG2)	5
			It makes more sense to me to say “Let’s go beyond the current hospitalisation and problem, and let’s talk about what is more long-term planning. Let us talk more broadly about the planning of a person’s health”. (FG2)	6
			Let’s make the discourse broader, let’s start dealing with what does not have to do with “now” but will have to do with it in a more holistic health-related discourse. (FG2)	7
			In the ideal world, I would like that each patient has this sheet, but it also matters when this sheet was compiled, because one thing is to do it now at twenty years old, and one thing is to do it at eighty. I will have a different idea depending on the conditions I will be. Surely you should have this sheet, even if finding everything written down sometimes is not so useful. (FG2)	8
		Explore patient’s understanding, expectations, wishes, and values	In the end what counts is respecting the patient’s wishes. (FG1)	9
			However, we must always take into account who we have in front of us and what we are doing. Even the fact that the institution imposes—and we impose it on ourselves, too—to have advance directives, or to have us expressing our choice for the patient. . . but in the end it is the choice of the individual, even for a ninety-four year old patient. The first thing is being there. (FG1)	10
			We need to focus on the patient’s objectives, not only on the therapies that we doctors want [. . .] What their values are, what their importance is, even if the patient, at that moment, is not in a phase of illness. So that they can say what they want, what their life is, and what they expect from an illness, and on this we decide a priori, before the illness, we decide what will be done if the illness arrives, without waiting. Every day [we should] have on our board, on our patient’s board, their goal—which is not ours, and which is detached from the medical act. (FG4)	11
			I say, ideally, we should start, in a situation where we have to understand how far to go with the treatments. The first thing is to understand what the patient has understood about their situation and thier pathology, so we can explain them a little, and get on their level, and only then get to the problem we have to solve together. (FG2)	12
			[We should] know the patient and what their expectations are, what they expect, and how they are aligned to the disease, this is the first thing. (FG1)	13
	The is			
		Involvement of the patient in the decision-maiking is futile until proven helpful	Clearly, if one finds that there may be value in starting this conversation, one does start it. If, on the contrary, we lack a reason to start this conversation, one avoids it. (FG2)	14
			I also think about the imminence. . . I mean, whether it is useful to talk about it as soon as possible with a patient with respiratory distress and who might shortly have an arrest. If, on the other hand, it is a patient who has a team following them, and is expected to be taken care of, then one can wait with common sense. (FG3)	15
	Justifications			
		Presumed lack of capacity	There are decisions that patients are not able to make because only a doctor can understand what the consequences for the patient might be. And so we risk to overtreat and do futile treatments. (FG1)	16
			If they don’t get there, it is useless. And at that moment we need to talk to the family doctor and share the choice with them, and if they don’t have the tools, the education, at most the choice has to be imposed and then shared. We tell them that we have chosen not to resuscitate them because the quality of life that is envisaged is not vaguely comparable to that of before, and then we refine it with the relatives. Then it does not mean that we will not treat them, on the contrary! The care is assured until resuscitation, which in that case is not indicated. (FG2)	17
			If one has very clear ideas, it is very easy, and no time is wasted. If, on the other hand, you have to start from scratch and there are no tools, there is no point in wasting time and one has to impose oneself and give the indication, perhaps sharing it with the family doctor if the choice is a bit borderline. (FG2)	18
			People wouldn’t understand it, they don’t have the tools to understand it in a context that seems totally unconnected to them. It’s different in the hospital in Zurich where they are asked "do you want to be resuscitated or not?", there’s a different culture, so you expect to be asked [that question]. (FG2)	19
			It’s so bad to leave the decision in the hands of a 90-year-old where they don’t even know what I’m talking about, they can’t even imagine. Where they tell me that they want us to do everything for them. In that case, I learned to take the relatives aside, and to explain to them what resuscitation means to a patient of that age, and how they would come out of it [CPR], and to discuss with them that from a medical point of view we would end it there, and if they didn’t agree they could still oppose the decision. My chief physician at the time used to end it like this, and [say] that if they didn’t agree, as he had the task of not harming the patient, they could transfer [the patient] to another hospital, but that he would not resuscitate a 90-year-old man in his hospital. [. . .] Sometimes you can discuss the therapeutic process but other times you realise that this discussion cannot even be carried on because it’s too difficult and it’s a decision that they wouldn’t be able to make, so you have to look for the relatives. (FG4)	20
			Perhaps, at certain times, the patient is unable to decide for themselves, and therefore there is no point in discussing this with the patient if it is not possible. (FG1)	21
			There is a whole sphere that we don’t share with the patient. . . We evaluate whether to tell the patient because maybe there is no real repercussion for them. If this patient ha sa cardiac arrest, I don’t resuscitate them, and they won’t notice. For example, with a demented patient. (FG2)	22
		Poor prognosis	It also happens in everyday hospital life not to discuss the advance directives with the patient. It has happened to all of us to say “OK, this is a patient who, if they have a complication, is not resuscitated, by medical choice, because he has a number of impairments and pathologies that would not give them a chance of a good recovery”. And so normally you inform them that you established this attitude of care, but we do not always discuss it with the patient. (FG1)	23
		Young age or good health	It is not that we discuss with all patients the advance directives, for example if it is a young patient, in good health and comes for something that does not involve any particular consequences. Whereas if a patient is really compromised, but also relatively young, then yes [we discuss it]. (FG1)	24
			With a healthy patient it may be difficult to deal with such talk (FG4)	25
		Need to ration limited resources	Here perhaps I have experienced different dynamics in these two years, we are talking about 16 months of Covid where the patient was admitted in the morning and by the evening you had to talk to them and not even ask for advance directives or what they would have wanted, because fate imposed itself violently. (FG1)	26
		Desire to avoid scaring the patient	It’s really pointless because you know the situation is so bad. . . Why scare them further? (FG1)	27
			With the 18-year-old coming in for a fracture, it is not discussed in the emergency room, and it is not done because it can become counterproductive and create anxiety. If done wrong, it is better not to do it [the conversation] in certain cases. (FG3)	28
			If they have never thought about it and they come to the emergency room, we don’t have time to have a good discussion, we just create anxiety in the patient. So in that case it makes sense, and it is not bad that we have to make the decision [for them], we help the patient and the family to take the responsibility. We orient the discussion and give advice on what we think might be the best solution for the patient. (FG2)	29
		Desire to preserve patient’s hope and the therapeutic alliance	Perhaps it is because of a fear on the part of the physician to extinguish the patient’s hope in treatment and in the possibility of being able to do something about their illness. Even if the physician is well aware of the fact that a disease is chronic and, as per its history, will be worse and worse, and that this worsening will not offer any possibility of treatment. They may find it hard to extinguish [the patient’s] hope in this way, hard to tell the patient the way things really are. It is not uncommon for us to observe people who remain incredulous when their disease is so advanced that it precludes short-term survival. In my opinion this holds back a little bit the physicians, this fear of losing the therapeutic alliance with the patient. (FG4)	30
			My reflection is on how not to take away the patient’s hope. (FG1)	31
		Old age combined with the presence of co-morbidities	It has happened to me so many times not to talk about it because the patient is 90 years old and is hospitalised for a trivial urinary tract infection but you still have to include the advance directives in Geco [the electronic health record] and you say “he is 90 years old, he has several pathologies”. . . And so you put the label “REA NO”. Here maybe the patient is lucid and oriented, but you don’t discuss it not because the situation is dramatic but because of all the comorbidities and the situation. (FG1)	32
			When there is a very old patient I believe in the medical decision and if the patient is able to understand it is communicated. If not, it is not. (FG4)	33
		Lack of independence	I remember when X was my supervisor, he took a scale to know the level of independence of the patient and with that we could write the “REA NO” instruction, because the patient was not independent enough. There was a whole scale to help make a decision, it would be nice to have a document that we could look at. [. . .] [a document] that tries to quantify in an objective way. (FG3)	34
	Solutions			
		Define the many hopes patients may have	It becomes almost easier in that area to enter into the issue of quality, to concern oneself with the patient’s values and wishes, which then become the sole objective. If one took this step earlier, I would not believe that the therapeutic alliance and hope would be altered. (FG4)	35
			We should define this hope. Is it a hope of recovery, hope of having a life as before, hope to be able to eat ice cream again? It really depends on the type of hope. (FG4)	36
			[We should] re-establish the therapeutic alliance by re-establishing the goal according to the current condition, informing them that we are there and that something can always be done with the goal perhaps no longer to heal and return to life as before, but with the goal of living an acceptable quality of life. Reducing the objective together with the patient and informing them of their condition, where they can inform themselves by creating a therapeutic alliance between patient and doctor. (FG4)	37
			Planning with the patient helps to give a little more hope. Instead, communicating a diagnosis, proposing possibilities to them with the hope of achieving something, we do not know if the first thing that goes wrong will restore hope. Planning with them what they expect and setting goals that we know are achievable for them, and not setting too high a goal allows us to work with the patient and work with them. It’s certainly not something you can do in a half-hour consultation where you often don’t even know the person. (FG4)	38
		Improve public awareness of ACP and AD	In the ideal world, it is not only the responsibility of doctors but also of society such as the willingness to donate organs. There are always many media campaigns and people know what it means. Instead, there is no campaign that says “Do you know the advance directives? Talk to your doctor!” (FG1)	39
			I also think that the population should be educated a bit on this subject because they don’t talk about it. When we meet them [patients], even with a proper setting, and aks them what they want to do, they don’t expect it, they never expect it. It’s really a subject that’s not talked about and it’s a subject where everyone gets surprised when it’s addressed. "What are advance directives? Never heard of them!” and there it’s already too late to discuss. (FG3)	40
		Breaking the taboo of talking about death	The world I would like. . . I see a world where acceptance of death is part of the directive form [in the electronic health records] because we live in a world where there is an inherent fear of death. This is part of the obstacle for us. When one gets used to these issues they become easier to deal with. (FG2)	41
Communication				
	The ought			
		Find the right place, time, and interlocutors before conversation	Of course it should not be done in a room with four other patients. You have to find a secluded place or a quiet room where no one will disturbe you. You leave the phone with your colleague and ask the nurse not to disturb you unless it is strictly necessary. (FG1)	42
			Any discussion should happen in an appropriate place. (FG2)	43
			In my opinion, the first thing is the timing and the appropriate setting. A discussion cannot be held at the time of the visit, you really need an appropriate place and time. (FG3)	44
			People who are with the patient, so family members or a friend or the patient alone or even the nurse who knows the patient [should be there]. (FG3)	45
			I would also involve the family doctor, because when we are in the hospital we have known the patient for a maximum of two weeks, and we must not forget that there is a family physician. (FG3)	46
			Also the number of people can be important to the patient. (FG3)	47
			A selected group that knows the patient’s life, who has been treating them, or who has an idea of their needs and knows the family members. (FG3)	48
		Establish rapport before conversation	I would rather wait a few days so that there is more of a relationship of trust there, and then the patient may ask “doctor, what would you do?”. And that makes things much easier. (FG1)	49
			For me it is very important that a therapeutic relationship has already been established with the patient. (FG3)	50
		Neutral, factual and non-leading information	The main thing for me is that the patient is aware and knows a little bit about what is behind it. (FG1)	51
			Do not ask “do you want to be resuscitated” because of course the answer is “yes”! (FG1)	52
			We should never get involved in the decision. We can explain to them, we can reassure them, but the important thing is never to influence them. (FG1)	53
			One must remain neutral. Explain things correctly. (FG1)	54
			We should be aware that we have the responsibility, often the task, to guide this choice. (FG2)	
			We used to say that [the patient] is currently hospitalised for something that should not give any complications and put life at risk, but we cannot predict anything, so we wanted to get to know them a little. So we would start with the patient to see what they understood, what their normal living conditions were like, how they lived at home, what they expected and how they didn’t want to live. And together with them, it was no longer paternalism, we would put all the options with our advice and what we could discuss together. (FG4)	55
		Inform that DNR does not preclude optimal care or treatment of the underlying condition	It must be well explained what resuscitation entails. The message should not be that if something serious happens we do nothing and stand by and watch. They must understand what resuscitation entails. (FG1)	56
			They may think we just let them die like that, but that is simply not what comes next. (FG1)	57
			It is frustrating that they may think we want to abandon them. (FG2)	58
		Stress that the decisions are not irreversible and can be adapted across conditions	What also stops the patient and the families a bit is the feeling of irreversibility of the choice. The choice is made and from that moment there can be no more discussion. It happened to me, that I had to fight with my colleagues, because I had a patient who was quite ill to the point that in fact it justified admission to the intensive care unit. But [the patient] had been admitted in March with Covid and there they had decided that if the patient had deteriorated they would not have transferred the patient to the intensive care unit and would have set up palliative care. And therefore the colleagues said that it was “tube no”, “resuscitation no” and that the patient had to be put in palliative care. While here we were in a situation of sepsis, and he had recovered very well after Covid; and yet, this decision was set in stone and every now and then I think that this could be a possible stumbling block to this discussion. Nobody wants to make this decision that cannot be changed and that will remain forever. (FG4)	59
		Keep the conversation open	Sometimes there are forms or diagrams that can be useful, but in others the relationship counts a lot. Sometimes patients arrive in the emergency department with such detailed directives that they contradict each other and are difficult to understand, whereas a discussion where a multifaceted but more coherent sense prevails would be more appropriate, avoiding the “yes” and “no” that is too detailed and confusing. (FG3)	60
		Consider the larger social context of the patient	I think it is also important to consider the burden not only for the patient but also for the social entourage around the patient. (FG3)	61
		Use words that communicate presence	There is precisely the advice to never be alone, even in communicating the news, not because you are not capable but because you “carry it together”. The other important thing we have to think about is the words we use because the patient always perceives if we are participating, if we really are there with him and if we really are there to help him. (FG1)	62
		Tailor the communication style to the patient	You also have to adapt to the moment, it’s not that there is a right formula for everyone, just how you get feedback from relatives and family members. Because you can’t use the same words with everyone, sometimes you have to be as sweet and empathetic as possible while sometimes you need to use the bluntest words for the message to get through. (FG1)	63
		Show humility	I think it is also important to be humble [. . .] Finally we talk about death and life and the patient is not just a patient and has a whole life before they were ill. (FG3)	64
		Embrace the conversation with courage and truthfullness	And so one really has to take the courage sometimes to have this talk. It is often the case that those treating chronic illnesses find it hard to say that the illness will not get better but will only get worse, and so it must be decided now that this is going to be their future. And they find themselves talking about it with complete strangers. (FG4)	65
			You shouldn’t say things you don’t know. . . If you get a specific question from the patient or family member, I would say “Look, I don’t know this thing exactly, but I’ll check.” (FG1)	66
		Sum up everything at the end	At the end, I have to summarise to the patient what has happened to that point we have arrived at, and then we talk frankly. (FG1)	67
	The is			
		An inevitably nudging conversation	When you are there and list all the frightening consequences, you have already made a suggestion. I don’t explain to a 30-year-old that if I resuscitate them they risk being intubated for months, with permanent brain damage. Unfortunately, very consciously, the way we explain the consequences of resuscitation influences the patient’s choice. In the end, it is not so bad because we are always the ones making a decision from the medical point of view, but there we are sharing it with him and the patient is also convinced that it is their decision. So that leaves the patient serene, it leaves us serene and that is the goal of it all, to make the shared decision. (FG1)	68
			It is us who ultimately make the family member choose what we would have done, so we have to acquire this awareness that we are the ones who decide. Depending on how I present the possible outcome of resuscitation or admission to the intensive care unit, or how I present the development, I end up putting words in the mouth of the family members who tell me not to do it. In the ideal world, we are all aware of this and that we are driving a choice. I have had discussions with clinic leader colleagues about these issues and just the way it was presented what CPR means, you are actually driving a choice. For example, to say “yes, we’re going to stand there, two pushes on the chest and maybe you’ll make it” is one thing, to say “it’s very violent stuff, we’re going to break your ribs, most patients don’t survive or you’re going to be a “vegetable” [in a vegeattive state], I’m already making you choose! We should be aware of this role and power we have, particularly when we lead a discussion about advance directives in an urgent context. (FG2)	69
			More and more choices are left to the patient, but these choices are. . . They go in a direction that is given by the physician. This is something which we inherit from Anglo-Saxon countries where we have to protect ourselves more and more with informed consent. So it seems to me that we inform patients a lot or pretend to inform them, making them take the decision, whereas it is obviously made by the physician. (FG4)	70
			We take a big right when we hide something big from the patient. We do not have the right to do so even legally. We have to tell the patient how things are. Sometimes, with the pressure of family members, there is a tendency to play things down. (FG4)	71
			Even I, when they ask me what I would do, give an answer. All of us, when talking about the consequences, are influencing the patient’s choice, because it scares them, but of course the choice is theirs. By listing all the complications we are directing them. (FG1)	72
		Purely factual information	This is the problem, because I realise that as a result of working hours and fatigue you have to give in on something, and you always start with a communication that remains only informative. (FG1)	73
		Inappropriate time and place	It has happened to me that in emergency situations you don’t have time and an optimal environment. You get a patient in the emergency room and you have to decide there. [. . .] Unfortunately, what I have experienced is not having the time to do things the way they are usually done on the ward. (FG1)	74
			I firmly believe that the hospital setting is not the ideal place to talk about advance directives. We can make it as good as possible but it is not the right place. (FG1)	75
			It is not always guaranteed. There are departments where there is no physical possibility to have such a discussion, not to be disturbed by the telephone.	76
			A key factor is time, to explain to the patient and to ask. Time is crucial. The problem with doing it on the ward is that there is no such time because it can take hours and is often impossible. (FG3)	77
		Physicians as the ’bad wolves’	If you’re the one talking about it, it sounds like you’re the bad guy and you’re talking about the unmentionable. I’m fed up with this. Because I spend a lot of time on it and in the end I don’t come up with anything, whereas if it’s a patient from the German-speaking Switzerland, it happens that they come to the hospital with their sheet with the advance directives already done. That’s the ideal, and I don’t expect it, but [I wish that] at least a 91-year-old has thought about it. Their family are suprprised and ask if it’s so serious and they have’t understood anything. (FG2)	78
			We really look like villains [to patients] and we create panic among people and relatives, when the question about resuscitation comes up. (FG2)	79
			I remember a bad experience with a colleague who accused me of mediocrity because I told the patient how things were, he told us internists that we were mediocre because we talked about these things with the patient when there was no need, and here the whole paternalistic attitude comes back, where it should no longer be like that, but only in part. In fact, when we reason about therapy and advance directives we lead the conversation towards our point of view. (FG4)	80
	Justifications			
		Lack of time	In emergency situations there is no time and that is why we create these two situations of the advance directives: [one] in the emergency, and then [the other one] something that we could discuss at a non-urgent time, when the patient is there for something else and is not in an imminently dangerous situation. (FG2)	81
			That is in fact an organisational problem, a practical one indeed. Assistants on the ward have a hard life and never have time, even family conferences are a good thing but often you don’t have the time to devote to them and if not done well they can become a nuisance where you discuss the patient’s attitude, but that has little to do with it. (FG3)	82
		Work-related fatigue	Sometimes we are stressed and it is difficult. (FG3)	83
		A time- and resource-consuming task	It is easier to treat the disease and not the person and this takes much less time and energy, and it is easier because there is often a defined pathway. (FG 4)	84
		Lack of previous conversation with GP	We have patients with whom this has never been discussed. For example, I have a patient who has been to the hospital at least four times in the last year and I had her son on the phone, and the son told me that no one had ever spoken to her about this. So we are overwhelmed to address an issue that should be done routinely, always addressed in inpatient stays. (FG2)	85
			With respect to the quality of life discourse, it is really a very long discourse that we struggle to make in the hospital, especially since it is done 90% in the acute phase whereas it should be done in the stationary phase both with a healthy patient and one with a chronic disease. (FG 4)	86
		Adherence to the principle of non-maleficence	I would divide it into two aspects. One is that here is a lack of clarity on protocols, algorithms. In the sense that there are our superiors who say “OK, now it is the patient who decides”. Whereas on the other hand there is a reasoning of non-maleficence in the omission from the doctor, to give adequate advice to patients and to advise what is adequate and to take the patient down a recommended path which is often a “REA NO”. Often, one is caught between these two paths and there is a lack of clarity. [. . .] There is a lack of clarity about where we doctors place ourselves and then we have to push, or where we leave it to the patients to decide. Directives are different than a decision about amputating or removing an organ, because it has to do with whether or not the person exists. Why is it so different from another medical intervention? (FG2)	87
	Solutions			
		Timely communication training	It is not something you improvise. It’s something you have to learn how to do, there are communication techniques, there are communication strategies, there are also principles of law that you have to know and I think you need training. It should be part of the practice, before you start working, you have to, if we want to make some cultural change, you have to start fromwho has the power to direct families and patients. Sometimes it’s just a technical question on how to communicate and knowing what to say, what not to say, the phrases to use, the way to present oneself, or let someone speak. It is something you learn. (FG2)	88
		A dedicated person in the team	I don’t exclude having dedicated people and dedicated staff, someone who will do this not in an improvised way on my third day on the job. One of the things to propose to the management, to the hospital is to provide structured training for all the people. Intensive care medicine, emergency room, etc. (FG2)	89
			There should always be a figure who accompanies you, like a clinic leader or even a dedicated figure who comes, helps you along the way. If not, you really struggle. (FG3)	90
		Exposure to conversations and debriefing	It’s often a "handmade” thing, in the sense that you have to be exposed to these kinds of discussions. The first few times I participated and kept my mouth shut, I listened and I also needed to see several people do it to see what techniques one and the other used. “Okay, this sentence I like a lot, okay, this example I will use it”. Then at the end one builds up one’s own knowledge, of course first one has to have knowledge with a structured training but it is also important to have a boss who brings the assistant, even on the first day of work and he just stands there mute.[. . .] After the discussion, the person who had led the discussion, the conversation, would stop with me and we do a kind of debriefing where we analyse how the patient reacted, what we could say better. It is really a structured thing. It has to be kind of spread out in a structured way. (FG2)	91
		Involvement of GPs and specialists	It would be better if the patient discussed the advance directives with the family doctor because the patient needs time to metabolise the meaning of things, e.g. resuscitation. They need time to understand and metabolise. (FG1)	92
			It is easier to talk to family doctors, with whom they have a relationship of trust. (FG1)	93
			Anticipate, with a treating doctor who dares to broach the subject even when the patient is healthy because it is much easier that way. (FG1)	94
			When one arrives at the hospital in an acute situation those questions there can only frighten them. If they are asked in a context where they are not in an emergency it can allow an even more lucid decision. (FG1)	95
			If the topic is introduced by a trusted person, such as the attending physician, this could also facilitate. (FG1)	96
			If we want to have a proper discussion, it should be done by the treating doctor outside the acute. (FG2)	97
			It is best done with one’s own doctor and health personnel who know the patient and whom the patient trusts. (FG3)	98
		Reassure patients that the conversation is part of routine	You try to reassure the patient about the discussion we are going to have, because it is a discussion that usually creates a lot of fear in patients. Usually they are there for a problem that is not acute but that could precipitate. Then they immediately ask you “Why so serious?”. Then you start by saying, “we would like to talk to you about something, it’s an issue we deal with all the patients we hospitalise”. You really try to anticipate the fact that we are not in an emergency situation but that these are issues we deal with routinely in our activity. (FG1)	99
			I use this stratagem with patients that maybe you don’t know how it’s going to end so I tell them “these are the advance directives, maybe try talking to your mum about it” and I always ask if the treating doctor has ever talked about it, because you make the subject seem something natural, not something that has to be done only there and when you deal with it we are close to death. If you deal with it earlier it’s better. So you put it in a different light, even if it’s difficult, and if they asked me right now I would say “Oh my God”! (FG1)	100
		Reassure patients they are not alone	You would try to explain the situation and that always worked, telling them that we would explain the next steps and that they could stay with their loved one and that we would keep them informed. You frame the case, and maybe while the patient is in the CT scan you go out for a moment and talk to the family because the worst thing in these moments is not knowing, maybe you are sitting there, abandoned on a chair, thinking that it’s serious without having any information. (FG1)	101
		Assess patient’s interpretations of the information	We must also understand what it means for the person listening to us, I always like to say this. If we are called to treat them, we must neither heal them nor make them live, that is to say, we must accompany them in this choice. (FG1)	102
		Give the patient time to process the information	Then, if possible, allow the patient to reflect on the two things when we are not in an emergency setting. (FG2)	103
			[We should] ask questions, yes, but also try to wait. I have noticed from palliativists that they ask a question and then keep quiet, which I have never done, and after five or ten seconds the patient starts to open up. Here, don’t be in a hurry to get an answer, ask the question and leave time to answer. Also, you have to ask the question about how they are experiencing it, to also respect the other points such as empathy. In my opinion it’s a skill that helps, we always tend to give the patient a direction and they slowly open up. (FG3)	104
			[We should] let the patient have their say, to abandon that proactive aspect that characterises us as a profession, to ask the patient what they want. For example, I had a patient with recurring pneumonias with subsequent hospitalisation, and to the question their answer was always “I don’t know where I get these pneumonias”, and I asked them why they think they always get pneumonias. Then they answered that they are weaker than the others and therefore have a chronic lung disease, that’s how we approach questions because in this way we investigate what the patient’s level of awareness is and at the same time through questions and answers given by us or self-answers. The last thing I learnt in palliative care is that we should not always feel obliged to talk, we should not be afraid of the silences between doctor and patient. When we have to have this discussion we have to take our time because there are moments when the patient needs minutes of reflection and silence, we should not be afraid of silence and therefore have to say something. (FG4)	105
Structural causes				
	Patient-provider			
		Paternalism	Because we come from a medicine that until a few years ago was paternalistic in which the doctor said what had to be done for the good of the patient and that’s it, I think we still have a legacy. Then you could make a speech about the feeling of omnipotence of doctors that outside the tradition still remains. (FG4)	106
		Illness-focused approach	We are not so attentive to the needs of the patient because we, too often, focus on treating the disease and not on treating the person. (FG4)	107
	Hospital			
		Policy	What bothers me personally is that I always have to put a label on it, I don’t know if it happens to you too? Almost immediately, from the moment the patient comes in it is true that the patient can rush in but I am not like that sometimes, it becomes an automatic thing but the directives of a patient are not so trivial to have to decide in a short time, as we often do, because it is part of the things to do when a new patient comes in. (FG1)	108
	Society			
		The taboo around death	It is a cultural problem for me, around here the subject is not addressed. (FG2)	109
			There is a cultural aspect, I have never lived in a culture where it was easy to talk about death, I don’t know if there are any. Sometimes we are surprised to see elderly patients and they have never spoken about it. (FG2)	110
			There is a lack of a culture of the advance directives, there is the taboo of death and the talking about something that might happen, and the taking death into account as an event that is part of life. We have to confront what kind of culture we are facing. (FG2)	111
			I realise that this is not the rule in the world in which we live and where patients and their relatives have the taboo of death, which is why if a patient arrives in the emergency room we don’t go and discuss if a person has a urinary tract infection and needs an antibiotic, we don’t go and tell the family member “but if they have a cardiac arrest, what do we do?” It’s not something that culturally works, it’s heresy to some. (FG2)	112
		The sanctity of life	It was precisely in this context that I had found a cultural aspect that was linked to a component of religion, even if less and less practised, this Catholic component of the Southern Alps I had felt very strongly. As also a certain concept of the sacredness of life and therefore one could help lengthen it. I had really felt it from a socio-cultural and religious point of view. (FG4)	113
			Society nowadays no longer accepts that someone dies, let’s say that no one would have asked fifty years ago to resuscitate an 80-year-old whereas now they would want to live another thirty, without considering the quality of life. . . And one who is not in the field of medicine does not imagine the post resuscitation and therefore I find that time spent explaining, in simple words, the complications often helps to find a solution. (FG4)	114

### CPR/DNAR decision-making: A tension dominated by the presumption of futility

When asked what decision-making approach participants believed should characterize decisions on CPR/DNAR and advance care planning, more generally, they reported that, ideally, they ought to engage patients in a timely process to make decisions on future care plans, particularly anticipating the possibility of severe conditions and eliciting what treatment outcomes patients would consider acceptable for themselves (#1–7). One participant added that, ideally, this approach should culminate with the drafting of the patient’s own advance directives, although it was noted that their usefulness is limited as they may be too specific and apt to change over time as patient’s preferences change (#8). In addition, they reported that CPR/DNAR decision-making should place the patients and their wishes at its centre, independently from the patient’s age (#9–10), and physicians should aim to explore patient’s understanding, expectations, wishes, and values (#11–13).

However, participants reported that patient inclusion in the decision-making regarding CPR/DNAR is often believed to be pointless. Participants (particularly resident physicians) stated that patient inclusion is deemed pointless until proven valuable (#14), i.e., patients are not included by default, but there should be a good reason to do it (e.g., imminent cardiac arrest, #15). Our participants offered a number of justifications for this tension. These included patients’ presumed lack of capacity, mainly described as the inability to understand factual information (#16–22), poor prognosis (#23), young age or good health (#24–25), need to ration limited resources (#26), desire to avoid scaring the patient (#27–29), desire to preserve patient’s hope and the therapeutic alliance (#30–31), old age combined with the presence of co-morbidities, even if the patient is mentally sharp and has a good prognosis (#32–33), and lack of independence (#34).

To resolve this tension between the ideal and the actual decision-making approaches, participants proposed three main solutions. The first involves the patient-provider relationship and consists in physicians making an effort to define the many hopes patients may have beyond the simple goal of healing (#35–38). The second solution calls for institutions to increase public awareness of advance care planning and advance directives (#39–40) while the third calls for a societal change in which each of us contributes to break the taboo of talking about death (#41).

### CPR/DNAR conversations: A tension dominated by physicians’ inevitable role of choice architect

When asked to report on the features of the ideal conversation regarding CPR/DNAR, participants reported to have a clear idea of the architecture of such a conversation. They identified the need to find the right place, time, and interlocutors to join the conversation (#42–48) and to establish rapport with the patient *before* the discussion takes place (#49–50). *During* the conversation, they reported the need to provide neutral and factual information that does not lead but rather guides the patient (#51–55); inform patients and their families, in case of an unilateral DNAR order, that this does not preclude optimal care or treatment of the underlying condition (#56–58); stress that the decision is not irreversible and can be adapted across conditions, not only with patients and their families, but also among the care team (#59); keep the conversation open, avoiding the tendency to fit every answer to either a “yes” or “no” (#60); consider the larger social context of the patients and their emotional burden (#61); use words that communicate presence (#62); tailor the communication style to the patient (#63); show humility (#64); and embrace the conversation with courage and truthfulness (#65–66). *At the end* of the conversation, participants reported the need to sum up its content, particularly the process through which a CPR/DNAR decision was reached (#67).

At the same time, participants were aware that discussions with patients and/or relatives or authorized representatives will always be strongly influenced by physicians’ personal attitudes and preferences. In particular, they reported that such conversations tend to be inevitably leading conversations, since the way physicians present any information regarding CPR has the power to affect patients’ perceptions regarding this procedure (#68). Participants agreed that, ultimately, it is the physician who makes the decision for the patient, by shaping their “choice architecture” and later presenting it as made by the patient simply because it was eventually accepted (#69–70). This persuasive approach is carried out not only by choosing certain words over others but also purposely hiding certain information or minimizing the situation (#71–72). While some call for physicians to become aware of this role as “choice architect” and the power it intrinsically entails (#69) others see this role as a legitimate part of the shared decision-making approach, in which physicians paternalistically make a decision for patients and, by later communicating it, they make it a shared one (#68). Participants also reported that physicians end up solely presenting factual information (#73) and that the time and place of the conversation are often inappropriate (#74–77). Rather than the courageous and truthful persons they ought to be, physicians reported that they end up being perceived as the “bad wolves”, i.e., as heartless individuals that throw people in panic and despair whenever they engage patients in CPR/DNAR conversations (#78–80).

Our participants offered a number of justifications for this tension. These include lack of time (#81–82), fatigue and stress due to the demanding nature of their job (#83), the fact that CPR/DNAR conversations are a time- and resource-consuming task (#84), and lack of previous conversations on CPR/DNAR between patients and their general practitioners (GPs) (#85–86). One participant felt justified in purposely nudging patients to make them accept the physician’s decision, as he/she felt compelled to adhere to the principle of non-maleficence (#87). According to this participant, physicians know what is best for their patients and need to prevent them from making the wrong decisions by gently leading them.

To solve this tension, participants suggested to offer timely communication training to physicians (#88), have a dedicated communication professional in the team (#89–90), ensure physicians are exposed to as many CPR/DNAR conversations as possible and offer debriefing sessions (#91), increase involvement of GPs and specialists to promote these conversations outside of the hospital setting (#92–98), reassure patients and their families that the conversation is part of routine (#99–100) and that they are not alone (#101), assess patient’s interpretations of the information received (#102), and give the patient time to elaborate the information (#103–105).

### The structural causes of this twofold tension

The third theme refers to the structural causes of the tensions concerning CPR/DNAR decision-making and conversations. These include factors related to the relationship between patients and providers. On the one side, participants reported that a paternalistic approach is still present where physicians’ recommendation is absolute and not questioned, and that a feeling of power over patients is still widespread among physicians (#106). On the other side, they reported that a structural cause of this tension is a clinical approach that still pays excessive attention to treating the illness rather than the person (#107). At a hospital policy level, participants complained that the current regulations urging physicians to include a code status as soon as the patient is admitted place pressure on them and demand them to rapidly execute a task that would instead need extensive time to be properly performed (#108). At a societal level, two main bottlenecks thought to have both cultural and religious roots are described as slowing down progress regarding CPR/DNAR decisions and conversations. The first one is the taboo around death, a topic that is consistently avoided and which participants said they are afraid of discussing (#109–112). The second is the widespread idea of the sanctity of life, according to which we ought to extend life as long as possible using any means we have (#113–114).

## Discussion

The aim of this study was to qualitatively explore physician-reported CPR/DNAR decision-making approaches and CPR/DNAR conversations with patients hospitalized in the internal medicine wards of the four main public hospitals in Ticino. We found that CPR/DNAR decisions were dominated by the belief that patient inclusion is often pointless, even though participants had structured views on what would make a desirable decision-making approach. Moreover, we found that participants were aware that most CPR/DNAR conversations are characterized by a nudging communicative approach where the physician pushes patients to his/her recommendation, despite the belief that conversations should be as neutral as possible. Finally, participants identified structural causes to the previous two tensions that go beyond the patient-physician relationship. In the next paragraphs, we contextualize and interpret our findings, and consider their implications in light of the study limitations.

A first finding is the tension between a desired shared decision-making approach and the belief that patient involvement in CPR/DNAR decisions is often pointless, particularly when patients are deemed as lacking decision-making capacity. This finding, which echoes previous evidence that most physicians desire patient participation in the decision-making process and yet patients are often excluded [27], suggests two beliefs and a possible ex post rationalization behavior. The first belief is that patient involvement in the decision-making process can only be justified when CPR has an acceptable predicted success rate; if CPR offers little or no likelihood of benefit, patient involvement in the decision-making process offers little or no likelihood of benefit either and, therefore, there is no need to negotiate, and a unilateral DNAR is justified. This points to a pragmatic idea of value attributed to patient involvement: since it will not change the decision, there is no point in involving patients. That physicians’ own preferences may predominate in the CPR/DNAR decision-making process for their patients, and that they may not want their participation or believe that it is not necessary are both well documented [28–30]. Such a decision-making approach neglects two important elements. First, a decision to withhold CPR without knowing or even contrary to the patient’s or surrogate decision-makers’ wishes may only be justified in cases when CPR cannot reasonably be expected to yield the intended clinical benefit or achieve agreed-on goals for care, or when acute care facilities exceed functional capacity and triage decisions become necessary [17]. Even in such cases, competing concerns (e.g., risks of litigation) should be acknowledged by physicians. However, a unilateral DNAR is likely to be an ethical lapse: the decision may instead be driven by physicians’ perception that it is extremely difficult to involve patients in this choice, or by the fact that they do not “believe” that CPR is worth trying. Even when it becomes ethically justifiable to unilaterally make a decision to withhold CPR, patients still have an ethical and legal right to be informed about their code status and clinicians’ medical opinion and advice, and involved in other, more or less related decisions [31]. In addition, circumstances may change that make it necessary to review the CPR/DNAR status with patient input. The second element is that decisions regarding whether it is “worthwhile” to resuscitate represent, in the majority of cases, a value judgement rather than a matter of physiological probability, therefore any decisions regarding patient involvement in their CPR/DNAR decision-making will also constitute a judgment based upon a particular set of values that may contradict those of the patient affected [32]. There is wide consensus that it is extremely problematic to define the concept of futility in a medical context [33]. When recommending against CPR, physicians should approach such patients or their surrogate decision-makers with a presumption against providing CPR but also remain attentive when discussing the patient’s values and goals for unique factors and concerns that might make CPR unusually beneficial [34]. The second belief suggested by our participants’ reports is that decisions on whether to involve patients in the decision-making regarding their CPR/DNAR should be exclusively based on decision-making capacity criteria [35]. First, some have expressed concerns that these criteria are excessively “cognitive” and do not sufficiently attend to noncognitive aspects of choice and more demanding autonomy conditions relating to authenticity, narrative coherence, patient values, or the impact of volition, motivation, and affect or emotions (such as the emotional capacity to care) [36, 37]. Second, patients are generally presumed to have decision-making capacity, but the threshold (which should be that of ordinary freedom) tends to be shifted in opportunistic ways. A patient’s decision-making capacity may come under question when distortions in thinking and understanding due to a significant impairment of mental abilities are identified or suspected [38]. Only in such cases an assessment is warranted, and its results and underlying arguments need to be appropriately documented and made available to the patient, or the patient’s representative, on request [38]. However, dismissing patients as lacking capacity (e.g., because they struggle to understand factual information, as many participants of this study reported), may be the symptom of a post-rationalization behavior to legitimize *a posteriori* unilateral CPR/DNAR decisions taken by the care team. Physicians may ascribe incapacity because they see the possibility or even the likelihood of discovering disagreement between a patient’s preference and their recommendations/perspectives, and may feel ill-suited to handle such a conflict. According to the AMA, “physicians should engage patients whose capacity is impaired in decisions involving their own care to the greatest extent possible, including when the patient has previously designated a surrogate to make decisions on his or her behalf” [39]. In Switzerland, this is not only an ethical but also a legal requirement (Art. 377 of the Swiss Civil Code) [40]. Our participants were aware of these ethical and legal requirements, and many showed to be engaged with an active process of reflection on not only the reasons why, in practice, this is hardly met, but also possible solutions to resolve this tension. This finding does not surprise. Contrary to the rest of Switzerland, the Canton of Ticino borders with Italy, from which it is heavily culturally influenced. Paternalistic attitudes based on the therapeutic privilege are widespread [41]. CPR/DNAR discussion rates in this Canton are likely to be more analogous to rates detected in Italy than in German- and French-speaking Swiss Cantons [42].

A second finding is the reported tension between a desired non-manipulative conversation and the actual, leading communication style adopted by participants. This finding echoes the results of previous studies which found that, despite thoroughly trained on the importance of and ways to respect patients’ autonomy, physicians unintentionally resorted to strategic forms of communication leading patients to opt for physicians’ preferences [43, 44]. This result points to two facts. The first is the distinction between persuasion, nudging and manipulation. Persuasion refers to “believing in something through the merit of reasons another person advances”, and it is influenced by appeals to cognition rather than emotion [45], while manipulation refers to altering the quantity, quality, manner and relevance of the information presented to increase the effectiveness of a message in order to deceive or mislead the interlocutor [46]. The first may represent a valid support to patient autonomy, while the second a source of autonomy deprivation. Our participants reported to engage in a nudging communication. Nudging refers to intentionally structuring the presentation of options or decision-making environments to increase the frequency of desired choices without restricting free choice [47]. Scholars disagree on what would ethically justify nudging patients towards the decision that the physician believes to be the most desirable one. Some argue that nudging could amount to manipulation and improper exploitation of cognitive weaknesses [48–50], while others defend this practice as a form of “libertarian paternalism” that is not blind to cognitive biases in our judgments, and that in fact tries to “influence choices in a way that will make choosers better off, as judged by themselves”, by preserving freedom of choice and supporting moral agency [47, 51, 52]. Similar to all interventions that seek to direct behaviour towards certain ends, our participants were aware that nudging is problematic as it implies that physicians may impose their own judgment on what is beneficial to the patient, and may fail to address the *reasons why* CPR/DNAR may be the right thing for the patients. According to the SAMS guidelines on decisions on CPR, since discussions with patients and/or surrogate decision-makers will always also be influenced by personal attitudes and preferences, it is essential for physicians to be self-critical and aware of and disclose one’s own position [17]. Interestingly, our participants queried themselves against not only what ends they want to achieve (i.e., avoid nudging patients towards their personal preferences) but also what persons they want to be (i.e., the courageous, honest physician who guides patients in their decision-making without concealing or distorting the information on CPR). Such reflection shows that participants’ engagement of values and virtues was a central matter in their perspectives on CPR/DNAR conversation: who they are, what they recognize as important, and what they can (sustainably) achieve in relation to their CPR/DNAR conversations with patients are fundamentally intertwined. Participants reported a desire to engage in good action but they also made explicit the commitments underlying those actions. This is also stressed by the fact that they recognized the multidimensionality of the issue of nudging in CPR/DNAR conversations and reflected on multi-level solutions (e.g., involving general practitioners, offering young physicians ad hoc training to such conversations, or mobilizing social change to break the taboo around death that may hamper such discussion). The second elements suggested by this finding is that our participants may believe that there is only one, single-purpose conversation to be held with patients for whom CPR would offer little or no benefit. While a conversation on patients’ CPR preferences should be initiated when CPR represents an option, other conversations should take place when a cardiac arrest is likely and CPR is not offered. In this case, neither nudging nor other persuasive communication approaches will be needed. Rather, physicians should reassure patients that they will be respected and helped achieve the goals they have in pursuing their “loyalties”. This can be achieved by asking questions on their understanding of where they are with their health, their worries for the future, their hopes and priorities, and what they are (not) willing to sacrifice [53].

Finally, our participants called for a societal transformation in which each of us contributes to break the taboo of talking about death. However, it should be a priority for physicians to be able to overcome this taboo in clinical conversations, and learn how to not shy away from having difficult but important conversations about death with patients and families. Physicians often are unsure how to initiate or proceed with end-of-life discussions, mostly because medical education regarding end-of-life protocols is suboptimal or even lacking.

Our results have a number of implications which we summarise in the following list.

- First, they stress the importance of ensuring that CPR/DNAR conversations are held in the appropriate environment, with a trustworthy professional, and before the acute event presents. Ideally, GPs should be in charge of this conversation as they are likely to have established rapport with patients and can initiate these conversations outside of an emergency situation within the context of advance care planning. A national working group co-led by the SAMS and the Federal Office of Public Health (FOPH) is currently working towards improving the framework conditions and quality standards of advance care planning in Switzerland [54].
- Our results also stress the importance of recognizing that communication interventions in clinical practice should be regarded as complex interventions that need to be learned in a manner similar to technical interventions (actions that providers take and procedures they implement to deliver treatment and care to patients).
- From a policy point of view, our findings highlighted the limitations of the code status form currently implemented in the hospital where our participants were employed. Physicians land on the form as soon as the patient’s electronic health record is opened, and are asked whether CPR is indicated or not. In its current shape, this form may constitute a barrier to start a CPR/DNAR conversation and may nudge physicians to unilaterally decide on the patient’s code status. A better question could be “What are patient’s wishes regarding CPR?”. If a DNAR is entered, a question should appear asking “Has a decision been discussed?”.

This study has a number of limitations. First of all, the recruitment of participants was difficult to the point that it required adapting the design from in-person to online data collection. We thus relied on a small number of participants. However, participants engaged in an active and lively conversation during all four focus group, and brought such a richness of perspectives that it was possible to identify substantial tensions in relation to the themes explored. Second, given the low response rate, our sample was not representative of the hospital population we targeted. Furthermore, given that participation was on a voluntary basis, it may be possible that those who participated were particularly interested in the subject or with a particular discontent or tension that other non-participating physicians would have not highlighted had they joined the study. However, the issues raised by our participants remain relevant and interesting to understand the tensions experienced by resident and staff physicians and how they resolve them. Third, because of the small sample and the specific study aim, we cannot claim we reached thematic saturation. However, we relied on the concept of information power to establish saturation rather than thematic redundancy [55]. Information power indicates that the more information the sample holds that is relevant for the study, the lower number of participants is needed. Fourth, as this was the first exploratory study on CPR/DNAR decision-making and conversations in Italian-speaking Switzerland, we decided to restrict our target population to resident and staff physicians and exclude nurses and other professionals who have a role in CPR/DNAR decision-making and discussions. Finally, caution must be exercised in extending the findings of this study to clinical specialties other than internal medicine, outpatient settings, and different geographical areas, where different perspective on CPR/DNAR decision-making and conversations may emerge.

## Conclusions

CPR/DNAR decisions are contextual and their symbolic meaning can be highly value laden. For these reasons, they represent judgments that cannot be validly determined without the patient’s contribution and that should be considered against the backdrop of a patient’s whole life story. Our results reiterate that making a CPR/DNAR decision requires initiating and holding potentially difficult conversations that even highly trained practitioners may feel ill-prepared for and uncomfortable with. Importantly, not only did our participants seem aware that imposing their views regarding CPR/DNAR on patients would be a case of abuse of power. They also queried themselves against the tension between acknowledging that patients can view their own good differently and choose otherwise than recommended, and the actual practice of respecting their views and wishes, as well as which virtues they want to embrace.

## Supporting information

S1 Table Interview grid. The table shows the main topics and subtopics addressed during the interview and the respective questions asked to participants.
(DOCX)
