# Supplementary material for: Obstacles to patient inclusion in CPR/DNAR decisions and challenging conversations: A qualitative study with internal medicine physicians in Southern Switzerland
Source: PLoS One. 2023 Mar 22;18(3):e0282270. doi: 10.1371/journal.pone.0282270 (PMC10032495; doi:10.1371/journal.pone.0282270)
Supplement: S1 Table — The table shows the main topics and subtopics addressed during the interview and the respective questions asked to participants. (DOCX) [file pone.0282270.s001.docx]

**S1 Table. Interview grid.** The table shows the main topics and subtopics addressed during the interview and the respective questions asked to participants.

| **Theme and subthemes** | **Mandatory questions** | **Probing/alternative questions** |
| --- | --- | --- |
| **Advance Care Planning (ACP)** |  |  |
| Definitions and understanding | How would you define ACP?  What comes to your mind when you hear the word ACP? | Have you ever heard of it?  If not, let’s reflect together on what it can possibly mean |
| Description | Could you list all procedures/treatments that should ideally be part of a discussion on ACP? | Think about the possible procedures/treatments that a patient may undergo during hospitalization in the internal medicine department – which are worth being discussed in advance? Why? |
| Priorities in addressing treatments with patients | In what order of priority would you address these procedures/treatments? Why? | Can you list them from the most important to the least important? Which would you skip, and which you would never skip? |
| Treatment’s aspects to address with patients | Which aspects of each treatment/procedure would you address (e.g., duration, invasiveness, etc…)? | What is worth discussing with the patient regarding each treatment/procedure? |
| Ethical justification for ACP | What is the goal of ACP, in your opinion? Which principle(s) do you think it respects? | The main principles of bioethics are respect for autonomy, non-maleficence, beneficence and justice. Which of these four principles does ACP seek to respect? |
| **Experience** | |  |
| Evaluation of the decision to initiate the discussion | We learn by experience how difficult it is to start conversations about CPR preferences and ICU interventions (especially out of context). Can you think of one time you felt this discussion needed to be initiated with one of your patients?  What made you think that “it was time” to start this discussion? What did you do? | Think about one time you had to decide whether or not to start a conversation about CPR preferences and ICU interventions. What did you do to make a decision on starting/not starting the conversation? If you decided to start it, what told you it was the right moment? |
| Barriers | If you did not initiate this discussion, why didn’t you do it? What prevented you from initiating it? | In case you did not start it, was there any particular reasons for this? |
| Emotions (moral distress) | If you did not initiate this discussion, how did you feel? | It is common among HCWs to experience distress when they believe something should be done with a patient (e.g. respecting his or her will), but this is not actually done. Has this ever happened to you? |
| Facilitators | If you did it, how did you initiate it? What facilitated starting this discussion? | Was there anything that helped you start the discussion? |
| Emotions | If you did it, how did you feel? | How did you feel after? Do you associate the discussion with any particular emotion? |
| Supervisor-mandated discussions | Can you think of one time you were asked by your supervisor to initiate this discussion with one of your patients? What did you do? If you did not do it, why was this the case? | Can you recall a time your supervisor asked you to initiate and/or continue a CPR/DNR discussion with a patient? What happened? |
| Ethical aspects | Is it always right to initiate such discussions (CPR, ICU interventions, etc.) with patients? Why? Why not? Under which circumstances wouldn’t it be right? | Are there cases in which you should *not* have such discussions? When? |
| Actual discussion | Once you have decided to ask your patient about his or her will regarding treatments, what happens? If you did start the discussion, how did you *start* it?  Can you describe one time when the discussion went particularly well and one time when it went particularly badly. What made it go well/what made it go wrong? | Can you describe how the conversation goes, once you start it? You can also pretend your colleague is your patient and you are having the ACP discussion with him/her. |
| Ideal discussion | In the ideal world, how should such discussions take place? Can you describe all steps (from a communication point of view)? What key information would you ask the patient? What would you base any variations on (what makes you say something in a certain way with a patient, and something else with another one)? | What would the ideal conversation look like? Can you simulate it? |
| **Discussion inclusion process** | |  |
| Facilitators | What can be done to ensure discussions are properly initiated? Once they are initiated, what facilitates a fruitful discussion? | Previously, you mentioned that X facilitated the discussion. What else can help, besides this? |
| Ideal skills | Which skills do you need to initiate a successful discussion? | Was there anything that helped you? (E.g., courage, preparing well before, watching others doing it) |
| Actors involved | Who should initiate these discussions? Who should follow-up with the patient? Should there be only one person/or more? Should there be someone trained in psychology or communication next to the physician? | Ideally, who should start this discussion? And what should happen after? |
|  | What is the role of the care team in initiating such discussions with patients? And what is its role in the follow-up? Who should be in charge?  When you have been asked to initiate the discussion, did you feel it was *your* responsibility to do it? If no, why? |  |
|  | What is the role of the family if such discussions? |  |
|  | What is the role of the surrogate? How is the surrogate identified? Who is responsible for identifying him/her? |  |
| Interprofessionalism | Are tasks split among members from different specialties or professions (not only physicians) when it comes to CPR/DNR discussions with patients? If so, how? If not, is there a particular reason? What should be done, ideally? | You work alongside senior physicians and nurses. Who does what? Who should do what? |
| Ideal timing | When is the best moment to initiate such discussions? Should they take place before admission? If so, when and with whom? | Previously, you recalled that you realized it was time to start the discussion one day. What made it the right time? |
| DNR module | What is your opinion on the DNR module on the electronic patient record? How easy is it to fill it? How easy is it to interpret what others have written in it? | What is your experience with the DNR module so far? What would you recommend changing? |
